# Job satisfaction among physicians and nurses involved in the management of multiple sclerosis: the role of happiness and meaning at work

**DOI:** 10.1007/s10072-021-05520-8

**Published:** 2021-08-07

**Authors:** Luca Negri, Sabina Cilia, Monica Falautano, Monica Grobberio, Claudia Niccolai, Marianna Pattini, Erika Pietrolongo, Maria Esmeralda Quartuccio, Rosa Gemma Viterbo, Beatrice Allegri, Maria Pia Amato, Miriam Benin, Giovanna De Luca, Claudio Gasperini, Eleonora Minacapelli, Francesco Patti, Maria Trojano, Marta Bassi

**Affiliations:** 1 Department of Pathophysiology, and Transplantation, Università degli Studi di Milano, Milan, Italy; 2 Multiple Sclerosis Centre, University Polyclinic Hospital G. Rodolico, Catania, Italy; 3 Department of Territorial Activities, Health District, Azienda Sanitaria Pronvinciale, Catania, Italy; 4 Psychology Service, Neurology and Neurorehabilitation Unit, San Raffaele Hospital, Milan, Italy; 5 Laboratory of Clinical Neuropsychology, Psychology Unit, ASST Lariana, Como, Italy; 6 IRCCS Fondazione Don Carlo Gnocchi, Florence, Italy; 7 Multiple Sclerosis Centre, Neurology Unit, Hospital of Vaio, Fidenza, Italy; 8 Multiple Sclerosis Centre, Neurology Unit, University Hospital SS Annunziata, University ‘G. D’Annunzio’, Chieti-Pescara, Italy; 9 Department of Neuroscience, San Camillo-Forlanini Hospital, Rome, Italy; 10 Department of Basic Medical Sciences, Neurosciences and Sense Organs, University of Bari, Bari, Italy; 11 Department of NEUROFARBA, University of Florence, Florence, Italy; 12 Department of Biomedical and Clinical Sciences L. Sacco, Università degli Studi di Milano, Milan, Italy

**Keywords:** Healthcare professionals, Multiple sclerosis, Job satisfaction, Job happiness, Job meaning

## Abstract

**Objective:**

Health professionals caring for persons with multiple sclerosis (MS) are faced with increasingly complex working conditions that can undermine their job satisfaction and the quality of their healthcare services. The aim of this study was to delve into health professionals’ job satisfaction by assessing the predictive role of happiness and meaning at work. Specifically, it was hypothesized that job meaning would moderate the relationship between job happiness and satisfaction.

**Methods:**

The study hypothesis was tested among 108 healthcare professionals (53 physicians and 55 nurses) working in eight MS centers in Italy. Participants were administered the Eudaimonic and Hedonic Happiness Investigation and the Job Satisfaction Questionnaire. Hierarchical regression analysis was performed to test the moderating role of job meaning between job happiness and satisfaction.

**Results:**

A significant interaction effect of job happiness and meaning on job satisfaction was identified for both physicians and nurses. When work was attributed low meaning, participants experiencing high job happiness were more satisfied with their work than those reporting low happiness; by contrast, when work was perceived as highly meaningful, participants’ levels of job happiness did not significantly contribute to job satisfaction.

**Conclusions:**

Focusing on the interplay between job happiness and meaning, findings bring forward practical suggestions for the preservation and promotion of job satisfaction among health professionals working with MS patients. Particularly, they suggest the need to strengthen those job-related aspects that may enhance job meaning, thus providing health professionals with significant reasons to persevere in their work in the face of daily challenges.

## Introduction

Health professionals caring for persons with multiple sclerosis (MS) are daily faced with relevant clinical challenges. MS is the most common immune-mediated neurodegenerative disease of the central nervous system in young adults, affecting approximately 2.3 million people worldwide [1]. Disease manifestations include physical, cognitive, and psychiatric symptoms following a highly heterogeneous course. Most patients initially present reversible episodes of neurological deficits; over time, however, deficits become permanent and disability advances. Management and treatment of MS patients require considerable commitment by a multi-disciplinary professional team. Long-term dedication can engender a sense of frustration and helplessness as patients’ illness progresses. Particularly, the constant exposure to patients’ suffering and distress, limited treatment effectiveness, no available cure, and working conditions often characterized by excessive workload, poor work autonomy, and inadequate staff can undermine health professionals’ daily work enjoyment and ultimately their job satisfaction [2].

Job satisfaction has been connected to relevant benefits for all workers, including health professionals [3]. Among physicians and nurses, higher job satisfaction is associated with lower stress and burnout levels, and higher organizational commitment [4–6]. Professionals’ job satisfaction can also benefit patients through the development of more effective doctor-patient communication [7] and perception of better care provision [8]. Last but not least, satisfaction with one’s work is related to job retention, with satisfied professionals being less likely to reduce working hours, quit their position, or opt for early retirement [9–11]. This issue is particularly relevant among professionals working with MS, as the number of patients steadily increases in parallel with growing shortage in staff supply [2]. Preservation and promotion of job satisfaction is thus warranted to both foster well-being at work among professionals and provide high-quality care to patients.

### The contribution of affect and meaning to job satisfaction

Based on Locke’s seminal definition of job satisfaction as “a pleasurable or positive emotional state resulting from an appraisal of one’s job or job experiences” (p 1300) [12], theoretical and practical work on job satisfaction has largely focused on affectivity as underlying workers’ judgments about job tasks, economic conditions, or organizational environment [13, 14]. However, research has shown that there is more to job satisfaction than positive feelings [15]. This is especially true in the healthcare sector, where negative experiences in managing patients’ conditions may prevail over positive ones [16]. Particularly, job meaning has been identified as a potential contributor to job satisfaction [17]. For work to be meaningful, individuals must be able to identify significance in their actions, reflecting personal values and beliefs [15]. This definition resonates with the concepts of job calling [18], often applied in relation to health professionals’ prosocial values and existential commitment to enhancing people’s health [16]. Initial research has identified job meaning as a powerful driver of career satisfaction [9] and a negative predictor of burnout [19] among physicians; meaning was also associated with higher job performance among nurses [20]. However, more studies are needed to investigate the role of this variable in relation to health professionals’ job satisfaction, considering its predominant affective denotation.

From the perspective of positive psychology [21], meaning and positive affectivity are not mutually exclusive concepts; they rather represent complementary dimensions of human experience. Particularly, in the work domain, Steger et al. have conceived of job meaning as a motivational variable interacting with positive affectivity [22]. In their study among white-collar workers, they identified a significant moderating effect of job meaning in the relationship between positive affective disposition and work engagement. Participants who perceived their work as highly meaningful engaged to a greater extent in their work, regardless of their affective disposition; in contrast, when work had low meaning, participants with high affective disposition engaged more in their work than those with a low one. Similarly, in another study among white-collar workers by Bassi et al. [23], a moderating effect of job meaning was identified in the relationship of job happiness with life satisfaction, autonomy, and environmental mastery. However, the observed interaction was in the opposite direction compared to Steger et al.’s findings [22]. When participants attached low meaning to their work, different levels of job happiness did not have a significant effect on the three outcome variables, whereas at high levels of job meaning, participants were more satisfied with their lives, and perceived more autonomy and environmental mastery if they reported high versus low job happiness.

These contrasting results may be related to the different operationalizations of positive affectivity in the two studies, or the different outcome variables under consideration. Nonetheless, they underscore the interaction effect between job meaning and positive affectivity, albeit with opposite implications. According to Steger et al.’s findings [22], high job meaning may have a “special compensation value” (p 357), in the face of workers’ low affective disposition, promoting endurance despite negative affective work experiences; based on Bassi et al.’s results [23], high job meaning may be a double-edged sword to those who report low happiness levels at work, suggesting the protective role of low job meaning in the face of low job happiness.

### Study aim

In light of the findings and research suggestions reported above, overall goal of the present study was to investigate job satisfaction among physicians and nurses involved in MS management in Italy. Specifically, we aimed at investigating the interaction of job meaning and positive affectivity in predicting job satisfaction, taking into account health professionals’ job role (physician and nurse). Positive affectivity was operationalized in terms of job happiness as in Bassi et al.’s study [23], as it was deemed to more closely reflect the hedonic component of job satisfaction entailed in Locke’s definition [12]. In line with previous studies [9, 14, 22, 23], we expected job happiness and job meaning to be positively related to job satisfaction among both physicians and nurses. In addition, we expected our findings to confirm the significant moderating role of job meaning in the relationship between job happiness and job satisfaction for both groups. Considering the mixed results previously obtained [22, 23], however, no hypothesis was specifically formulated on the type of interaction effect at high and low values of participants’ job meaning.

## Methods

### Participants and procedures

Participants in this cross-sectional study were physicians and nurses working at eight MS centers in Northern, Central, and Southern Italy, representing different geographical areas in the country. They were recruited before COVID-19 pandemic as part of a larger project investigating the well-being of persons with MS and their formal and informal caregivers. The study protocol was in line with the Declaration of Helsinki and was approved by local ethical committees.

Professionals were approached by a researcher who presented the study and asked them for voluntary participation. Upon acceptance, participants were invited to sign the informed consent form and were given a set of questionnaires they could complete in situ or at home, and then give back to the researcher after 1 week/10 days. Questionnaires, data coding, and storage were anonymous.

### Measures

Participants provided information on their age, gender, education, civil status, job role, and seniority. They further completed questionnaires assessing the variables of interest in this study. *Job happiness* and *job meaning* were measured with two items from the Eudaimonic and Hedonic Happiness Investigation [24]: Participants were asked to evaluate their level of happiness at work on a scale from 1 “extremely low” to 7 “extremely high,” and how meaningful work was for them on a scale from 1 “not meaningful at all” to 7 “extremely meaningful.” *Job* *satisfaction* was assessed with an item from the Job Satisfaction Questionnaire [25], asking participants to report their overall satisfaction with their job on a scale from 1 “extremely dissatisfied” to 10 “extremely satisfied.”

### Data analysis

Data were first screened for multivariate analysis requirements in terms of normality distribution and outliers. Records that did not meet the necessary assumptions were removed in order to ensure soundness and reliability of results. Descriptive statistics were calculated separately for physicians and nurses, and jointly for all professionals. Between-group comparisons were performed with *t* test and chi-square test. Correlations were computed between job happiness, meaning, and satisfaction. A hierarchical linear regression analysis was performed to test for the direct effects of job happiness and meaning on job satisfaction as well as the moderating effect of job meaning in the relationship between job happiness and job satisfaction. To account for participants’ profession, a dummy variable for job role (1 = physician; 0 = nurse) was entered in the regression, and included in the interaction terms. To reduce multicollinearity, job happiness and meaning were centered at their mean values prior to creating product terms. Significance of regression coefficients was estimated through bias-corrected 95% confidence intervals (CI) from 1000 bootstrapped samples in order to ensure the robustness of findings. Coefficients were significant if 0 was not contained within the intervals. Simple slopes were calculated for low and high job meaning values, respectively, corresponding to the 16th and 84th percentile of the job meaning distribution, as suggested by Hayes [26]. Slopes were then plotted and tested for significance through *t* test.

## Results

Researchers approached 56 physicians and 65 nurses. Of these, one physician and six nurses declined participation due to lack of time or interest. One nurse was excluded after data collection because of substantial missing information; three nurses and two physicians were also excluded due to violation of multivariate assumptions necessary for regression analysis. The final dataset comprised 53 physicians and 55 nurses (*N*_total_ = 108), respectively, 94.6% and 84.6% of the professionals originally contacted. Physicians comprised 45 neurologists, 4 neurologists in training, and 4 physiatrists. The majority of physicians (62.4%) and nurses (65.5%) preferred to complete the questionnaires at home rather than in situ.

Participants’ demographic and job characteristics are reported in Table 1. Most participants were women, in their forties, and married or cohabiting, with no significant differences between physicians and nurses. They had a mean job seniority of 17.17 years, with nurses reporting significantly longer job experience than physicians (*t*(106) = 3.60, *p* < 0.001).

**Table 1:** Participants’ demographic and job characteristics

	Health professionals (*N* = 108)	Physicians (*N* = 53)	Nurses (*N* = 55)
Age, *M* (SD)	42.46 (9.53)	41.08 (9.74)	43.80 (9.22)
Gender, *N* (%)			
Women	78 (72.2)	35 (66.0)	43 (78.2)
Men	30 (27.8)	18 (34.0)	12 (21.8)
Civil status, *N* (%)			
Single	12 (11.1)	8 (15.1)	4 (7.3)
Married or cohabiting	72 (66.7)	36 (67.9)	36 (65.5)
Engaged	14 (13.0)	6 (11.3)	8 (14.5)
Separated/divorced	8 (7.4)	3 (5.7)	5 (9.1)
Widowed	2 (1.9)	-	2 (3.6)
Job seniority (in years), *M* (SD)	17.17 (10.31)	13.72 (9.30)	20.49 (10.22)

Descriptive statistics and correlations among variables are reported in Table 2. On a range 1–10, both physicians and nurses reported average ratings of job satisfaction above the middle point. Compared with data collected from a large sample of Italian physicians (*N* = 254; mean/SD = 7.4/1.6) and nurses (*N* = 251; mean/SD = 7.0/1.8) using the same questionnaire [25], nurses in our study reported higher satisfaction levels (*t*(100.22) = 2.36, *p* = 0.02), whereas no significant differences were observed for physicians. On a scale range 1–7, both professional groups reported average scores of job happiness and meaning above the middle point. No previous studies collected data on health professionals’ job happiness and meaning levels with the same questionnaire; therefore, no comparative analysis could be conducted.

**Table 2:** Descriptive statistics and correlations among study variables

	Health professionals (*N* = 108)		Physicians (*N* = 53)		Nurses (*N* = 55)		(1)	(2)	(3)
	*M*	*SD*	*M*	*SD*	*M*	*SD*			
(1) Job happiness	5.20	1.00	4.87	0.92	5.53	0.98	-	.16	.45**
(2) Job meaning	6.04	0.82	5.91	0.84	6.16	0.79	.20	-	.28*
(3) Job satisfaction	7.55	1.28	7.58	1.20	7.51	1.36	.56***	.08	-

*Note*. Correlations for physicians are reported above the diagonal; correlations for nurses are reported below the diagonal

^***^p < .001; **p < .01; *p < .05

Comparisons between physicians and nurses detected a significant difference for job happiness, with nurses scoring higher than physicians (*t*(106) = 3.60, *p* < 0.001). Among physicians, job satisfaction correlated positively with job happiness and meaning; among nurses, it correlated positively with job happiness.

Prior to regression analysis, correlations were calculated between job happiness, meaning, satisfaction, and participants’ socio-demographic variables. Since no significant coefficients were identified, no control variables were added to the model. Participants’ job satisfaction was first regressed stepwise on job role, happiness, meaning, and their cross products (role x happiness; role x meaning; happiness x meaning; and role x happiness x meaning). The recommended minimal ratio of cases to model parameters (10:1) was met in our sample [27]. The overall model was significant and explained 35.8% of the variance of job satisfaction (*F*(7,100) = 7.981, *p* < 0.001). A significant positive effect was observed for job happiness (*p* = 0.001; 95% CI: 0.51;1.28), and a significant negative effect was identified for the interaction between job happiness and meaning (*p* = 0.002; 95% CI: − 0.88, − 0.01). However, job role and the 2-way and 3-way interaction terms including this variable did not provide any significant contribution to job satisfaction. For the sake of parsimony, another model was thus performed, removing job role and its cross products from analysis. As shown in Table 3, this model globally explained 28% of the variance of job satisfaction.

**Table 3:** Hierarchical regression analysis for job satisfaction

	95% CI							
	*B*	SE	*β*	Lower	Upper	Δ*F*	Δ*R*^2^	*R*^2^
Step 1						30.72	.225	.22***
Job happiness	.60**	.14	.47	.33	.88			
Step 2						.59	.004	.23
Job happiness	.59**	.14	.46	.31	.86			
Job meaning	.11	.13	.07	− .13	.37			
Step 3						7.52	.052	.28**
Job happiness	.64**	.13	.50	.38	.90			
Job meaning	.06	.12	.04	− .19	.30			
Job happiness x meaning	− .37*	.15	− .23	− .64	− .04			

*Note. B* = regression coefficients; *β* = standardized regression coefficients. Standard Errors (SE), and confidence intervals (CI) were based on 1000 bootstrap samples

^***^p < .001; **p < .01; *p < .05

**Fig. 1:**
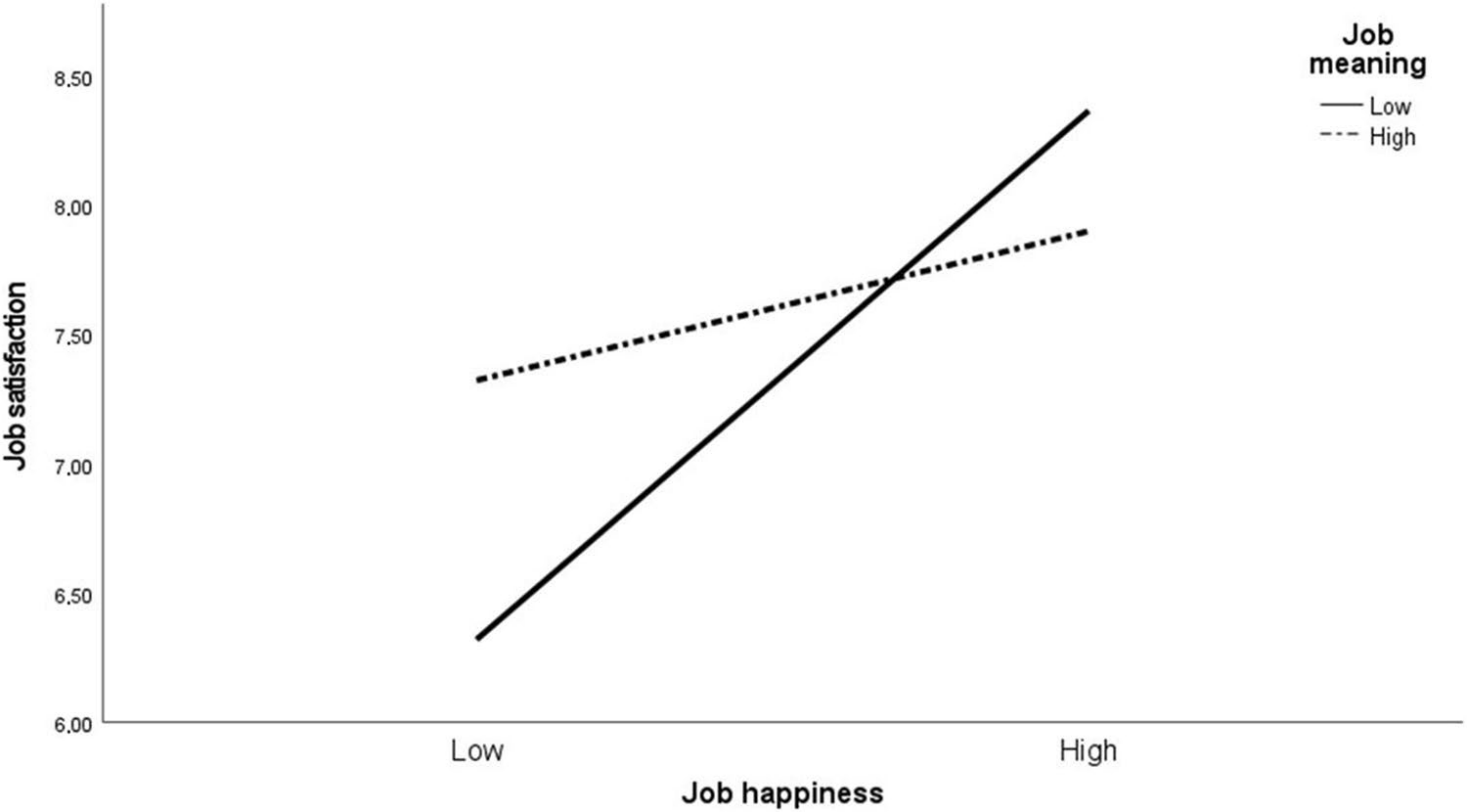
Interaction effect between job happiness and job meaning on job satisfaction

A significant positive effect for job happiness and a significant negative effect for the cross product between happiness and meaning were confirmed. Job happiness explained 22.5% of the model variance, while the interaction explained an additional 5.2%. The interaction was plotted in Fig. 1, illustrating how the relationship between job happiness and satisfaction differed at low and high values of job meaning. Only the simple slope for low job meaning was significant (*t*(104) = 5.302, *p* < 0.001), showing that levels of job happiness had different effects on satisfaction when participants attached low meaning to their work. Specifically, participants who reported low job meaning were more satisfied with their job when they reported high job happiness, and were less satisfied with it when they reported low happiness. By contrast, for participants attributing high meaning to their work, levels of job happiness—be they high or low—did not significantly contribute to job satisfaction.

## Discussion

To the best of our knowledge, this was the first study investigating the relationship between job happiness, meaning, and satisfaction among health professionals working with MS patients. While the bulk of research has examined well-being among MS patients and their informal caregivers [28, 29], few studies have specifically addressed formal caregivers [2, 9, 10], in spite of their central role in providing quality care [30].

Our findings revealed that study participants were globally satisfied with their job. Physicians’ satisfaction ratings were in line with those from previous studies among physicians with different specializations [25]. Considering that neurologists—who represented the largest group of doctors in our sample—were formerly found to report the lowest levels of job satisfaction among physicians [9], this finding supports the overall good level of satisfaction with work identified in our study. Among nurses, satisfaction ratings were even higher than those of registered nurses from previous studies [25], in line with international findings among MS-certified nurses [10]. Moreover, both physicians and nurses were globally happy with and found meaning in their work, reporting scores above the middle point of the measurement scales. Lack of previous data on health professionals’ job happiness and meaning levels collected with the same questionnaire prevented us from conducting comparisons, thus requiring further investigation. However, nurses in our study were happier with their job than physicians, underscoring nurses’ positive affective response to their practice [10].

Central to our investigation, a hierarchical regression analysis was conducted to examine the relationship between job happiness and meaning with participants’ job satisfaction. As expected, job role did not uniquely contribute to job satisfaction and did not moderate any relations between criterion and predictor variables, suggesting analogous relational patterns for both physicians and nurses. Contrary to expectations, a significant direct effect was identified for job happiness but not for job meaning. Particularly, increases in job happiness were associated with increases in job satisfaction among participants, underlining the affective component of job satisfaction reported in the literature [12–16].

While job happiness took precedence over meaning in directly contributing to professionals’ job satisfaction, however, job meaning proved to exert a significant moderating effect in the relationship between job happiness and satisfaction. In line with Steger et al. [22], simple slope analysis revealed that only the slope for low job meaning was significant, and not the one for high job meaning as in Bassi et al. [23]. When work was attributed low meaning, participants who reported high job happiness were more satisfied with their work than those who reported low happiness; in contrast, when work was perceived as highly meaningful, participants’ levels of job happiness did not significantly contribute to their job satisfaction. In line with Steger et al. [22], these findings thus lend support to the role of high job meaning in compensating for the negative affective experiences that may take place at work. According to Frankl [31], meaning can sustain a person even in the most difficult times, and suffering can be endured if there is a purpose to it. From this perspective, instead of relying solely on their affective experiences to achieve job satisfaction, health professionals with high job meaning may manage to tolerate frustrations occurring in their daily practice in the pursuit of a highly valued purpose associated with their work.

Interpretations stemming from present findings should be considered in light of some study limitations. The homogeneity of the sample, including physicians and nurses, restricts the generalization of findings. Although these professionals are predominantly involved in MS care, teams may also comprise physiotherapists, language therapists, and neuropsychologists: Sampling their job experience may thus provide more detailed information. In addition, data were only collected in Italy; considering the variety of healthcare systems around the world, international studies are needed to generalize our findings to other countries. Future studies could also involve professionals working with other types of pathologies, in order to assess whether the moderation of job meaning between job happiness and satisfaction applies across healthcare domains. Another limitation was that data were cross-sectional, thus precluding causal conclusions; longitudinal studies are needed to support present results. Finally, study variables—job satisfaction, happiness, and meaning—were measured with single items in order to contain survey length and respondents’ completion efforts. Such a procedure was adopted in many other studies [17], supporting the face validity of these items. Future studies can however benefit from employing multidimensional measures that could provide more articulated information on participants’ interpretation of these constructs.

In spite of these limitations, study findings bring forward some practical suggestions for the preservation and promotion of job satisfaction among health professionals working with MS patients. As both the incidence and prevalence of MS are increasing in Italy and worldwide [1], professionals are confronted with more complex working conditions which can undermine their job satisfaction, as well as job retention. Working conditions may also affect career choice among students in healthcare degrees, with relevant consequences in care provision to patients [9, 16]. In addition, the current changes in MS care delivery caused by the COVID-19 pandemic [32] may have potential long-term effects on professionals’ job satisfaction. To date, in spite of the heavy workload and high burnout incidence among healthcare staff working during the first pandemic wave, studies have identified consistently high levels of job satisfaction [33–35], stressing the need to monitor professionals’ well-being at work as the pandemic develops. Present findings suggest that job satisfaction could be enhanced by providing health professionals with opportunities for happiness with work. These may span across aspects that were previously identified as contributing to physicians’ and nurses’ job satisfaction, including higher remuneration, good relationship with colleagues and patients, increase in work autonomy, task diversification, professional growth, time efficiency, adequate staffing, as well as safety and protective personal equipment as highlighted during the pandemic [4, 9–11, 32, 34]. However, such a comprehensive intervention—though highly welcome—may not be realistic considering the dearth of resources and the challenges healthcare systems are facing worldwide. Based on the interplay between job happiness and meaning found in this study, a complementary approach to promoting job satisfaction could be focused on meaning. Intervention could prioritize strengthening those aspects that may enhance meaning in health professionals’ work, such as those related to self-actualization like professional growth and autonomy, and to patient-centered care like quality time and relations with patients [4, 15, 36]. Such a meaning-focused intervention would be in line with the prosocial values characterizing healthcare delivery [16], and could provide professionals with valuable reasons to persevere in the face of daily hassles and high emotional demands.

## Data Availability

Data are available from the corresponding author upon request.

## References

[1] Filippi M, Bar-Or A, Piehl F, Preziosa P, Solari A, Vukusic S, Rocca MA (2018). Multiple sclerosis. Nat Rev Dis Primers.

[2] Chesi P, Marini MG, Mancardi GL, Patti F (2020). Listening to the neurological teams for multiple sclerosis: the SMART project. Neurol Sci.

[3] Fischer JAV, Sousa-Poza A (2009). Does job satisfaction improve the health of workers? New evidence using panel data and objective measures of health. Health Econ.

[4] Khamisa N, Peltzer K, Ilic D, Oldenburg B (2016). Work related stress, burnout, job satisfaction and general health of nurses: a follow-up study. Int J Nurs Pract.

[5] Tziner A, Rabenu E, Radomski R, Belkin A (2015). Work stress and turnover intentions among hospital physicians. The mediating role of burnout and work satisfaction. J Work Organ Psychol.

[6] Saber DA (2014). Frontline registered nurse job satisfaction and predictors over three decades: a meta-analysis from 1980 to 2009. Nurs Outlook.

[7] Bensing JM, van den Brinnk-Muinen A, Boerma W, van Dulmen S (2013). The manifestation of job satisfaction in doctor-patient communication: a ten-country European study. Int J Person-Centered Med.

[8] Grembowski D, Paschane D, Diehr P, Katon W, Martin D, Patrick DL (2005). Managed care, physician job satisfaction, and the quality of primary care. J Gen Intern Med.

[9] Busis NA, Shanafelt TD, Keran CM, Levin KH, Schwarz HB, Molano JR, Vidic TR, Kass JS, Miyasaki JM, Sloan JA, Cascino TL (2017). Burnout, career satisfaction, and well-being among US neurologists, in 2016. Neurology.

[10] Gulick EE, Halper J, Costello K (2007). Job satisfaction among multiple sclerosis certified nurses. J Neurosci Nurs.

[11] Teixeira-Poit SM, Halpeern MT, Kane HL, Keating M, Olmsted M (2017). Factors influencing professional life satisfaction among neurologists. BMC Health Serv Res.

[12] Locke EA, Dunnette MD (1976). The nature and causes of job satisfaction. Handbook of industrial and organizational psychology.

[13] Fisher CD (2010). Happiness at work. Int J Manag Rev.

[14] Gurkova E, Cap J, Ziakova K, Duriskova M (2011). Job satisfaction and emotional subjective well-being among Slovak nurses. Int Nurs Rev.

[15] Steger M, Oades LG, Steger M, Delle Fave A, Passmore J (2017). Creating meaning and purpose at work. The Wiley Blackwell handbook of the psychology of positivity and strengths-based approaches at work.

[16] Wiesmann U, Oades LG, Steger M, Delle Fave A, Passmore J (2017). Well-being in health professionals: positive psychology at work. The Wiley Blackwell handbook of the psychology of positivity and strengths-based approaches at work.

[17] Bailey C, Yeoman R, Madden A, Thompson M, Kerridge G (2019). A review of the empirical literature on meaningful work: progress and research agenda. Hum Resour Dev Rev.

[18] Dik BJ, Duffy RD (2009). Calling and vocation at work: definition and prospects for research and practice. Couns Psychol.

[19] Ben-Itzhak S, Dvash J, Maor M, Rosenberg N, Halpern P (2015). Sense of meaning as a predictor of burnout in emergency physicians in Israel: a national survey. Clin Exp Emerg Med.

[20] Tong L (2018). Relationship between meaningful work and job performance in nurses. Int J Nurs Pract.

[21] Ryan RM, Deci EL (2001). On happiness and human potentials: a review of research on hedonic and eudaimonic well-being. Annu Rev Psychol.

[22] Steger M, Littman-Ovadia H, Miller M, Menger L, Rothmann S (2012). Engaging in work even when it is meaningless: positive affective disposition and meaningful work interact in relation to work engagement. J Career Assess.

[23] Bassi M, Bacher G, Negri L, Delle Fave A (2013). The contribution of job happiness and job meaning to the well-being of workers from thriving and failing companies. Appl Res Qual Life.

[24] Delle Fave A, Brdar I, Friere T, Vella-Brodrick D, Wissing MP (2011). The eudaimonic and hedonic components of happiness: qualitative and quantitative findings. Soc Indic Res.

[25] Steca P, Ripamonti C, Preti E, Monzani D (2008). La soddisfazione lavorativa dei professionisti della salute [Health professionals’ job satisfaction]. Psicologia della Salute.

[26] Hayes AF (2018). Introduction to mediation, moderation, and conditional process analysis: a regression-based approach.

[27] Cohen J, Cohen P, West SG (2002). Applied multiple regression/correlation analysis for the behavioral sciences.

[28] Gil-Gonzalez I, Martin-Rodriguez A, Conrad R, Perez-San-Gregorio MA (2020). Quality of life in adults with multiple sclerosis: a systemic review. BMJ Open.

[29] Maguire R, Maguire P (2020). Caregiver burden in multiple sclerosis: recent trends and future directions. Curr Neurol Neurosci Rep.

[30] Koudriavtseva T, Onesti E, Pestalozza IF, Sperduti I, Jandolo B (2012). The importance of physician-patient relationship for improvement of adherence to long-term therapy: data of a survey in a cohort of multiple sclerosis patients with mild and moderate disability. Neurol Sci.

[31] Frankl V (1963). Man’s search for meaning: an introduction to logotherapy.

[32] Morrison EH, Michtich K, Hersh CM (2021). How the COVID-19 pandemic has changed multiple sclerosis clinical practice: results of a nationwide provider survey. Mult Scler Relat Disord.

[33] Khalafallah AM, Lam S, Gami A, Dornbos DL, Sivakumar W, Johnson JN, Mukherjee D (2020). Burnout and career satisfaction among attending neurosurgeons during the COVID-19 pandemic. Clin Neurol Neurosurg.

[34] Savitsky B, Radomislensky I, Hendel T (2021). Nurses’ occupational satisfaction during Covid-19 pandemic. Appl Nurs Res.

[35] Giménez-Espert M, Prado-Gascó V, Soto-Rubio A (2020). Psychosocial risks, work engagement, and job satisfaction of nurses during COVID-19 pandemic. Front Public Health.

[36] Wong PT, Ivtzan I, Lomas T, Oades LG, Steger M, Delle Fave A, Passmore J (2017). Good work: the Meaning-Centered Approach (MCA). The Wiley Blackwell handbook of the psychology of positivity and strengths-based approaches at work.

